# Origin and significance of two pairs of head tentacles in the radiation of euthyneuran sea slugs and land snails

**DOI:** 10.1038/s41598-021-99172-5

**Published:** 2021-10-25

**Authors:** Bastian Brenzinger, Michael Schrödl, Yasunori Kano

**Affiliations:** 1 SNSB–Bavarian State Collection of Zoology, Münchhausenstr. 21, 81247 Munich, Germany; 2 Department of Marine Ecosystems Dynamics, Atmosphere and Ocean Research Institute, The University of Tokyo, 5-1-5 Kashiwanoha, Kashiwa, Chiba 277-8564 Japan; 3 Department Biology II, BioZentrum, Ludwig-Maximilians-Universität, Großhadernerstr. 2, 82152 Planegg-Martinsried, Germany; 4 SNSB–Bavarian State Collection of Paleontology and Geology, GeoBioCenter LMU, Richard-Wagner-Str. 10, 80333 Munich, Germany

**Keywords:** Zoology, Phylogenetics, Taxonomy

## Abstract

The gastropod infraclass Euthyneura comprises at least 30,000 species of snails and slugs, including nudibranch sea slugs, sea hares and garden snails, that flourish in various environments on earth. A unique morphological feature of Euthyneura is the presence of two pairs of sensory head tentacles with different shapes and functions: the anterior labial tentacles and the posterior rhinophores or eyestalks. Here we combine molecular phylogenetic and microanatomical evidence that suggests the two pairs of head tentacles have originated by splitting of the original single tentacle pair (with two parallel nerve cords in each tentacle) as seen in many other gastropods. Minute deep-sea snails of *Tjaernoeia* and *Parvaplustrum*, which in our phylogeny belonged to the euthyneurans’ sister group (new infraclass Mesoneura), have tentacles that are split along much of their lengths but associated nerves and epidermal sense organs are not as specialized as in Euthyneura. We suggest that further elaboration of cephalic sense organs in Euthyneura closely coincided with their ecological radiation and drastic modification of body plans. The monotypic family Parvaplustridae nov., superfamily Tjaernoeioidea nov. (Tjaernoeiidae + Parvaplustridae), and new major clade Tetratentaculata nov. (Mesoneura nov. + Euthyneura) are also proposed based on their phylogenetic relationships and shared morphological traits.

## Introduction

**Figure 1:**
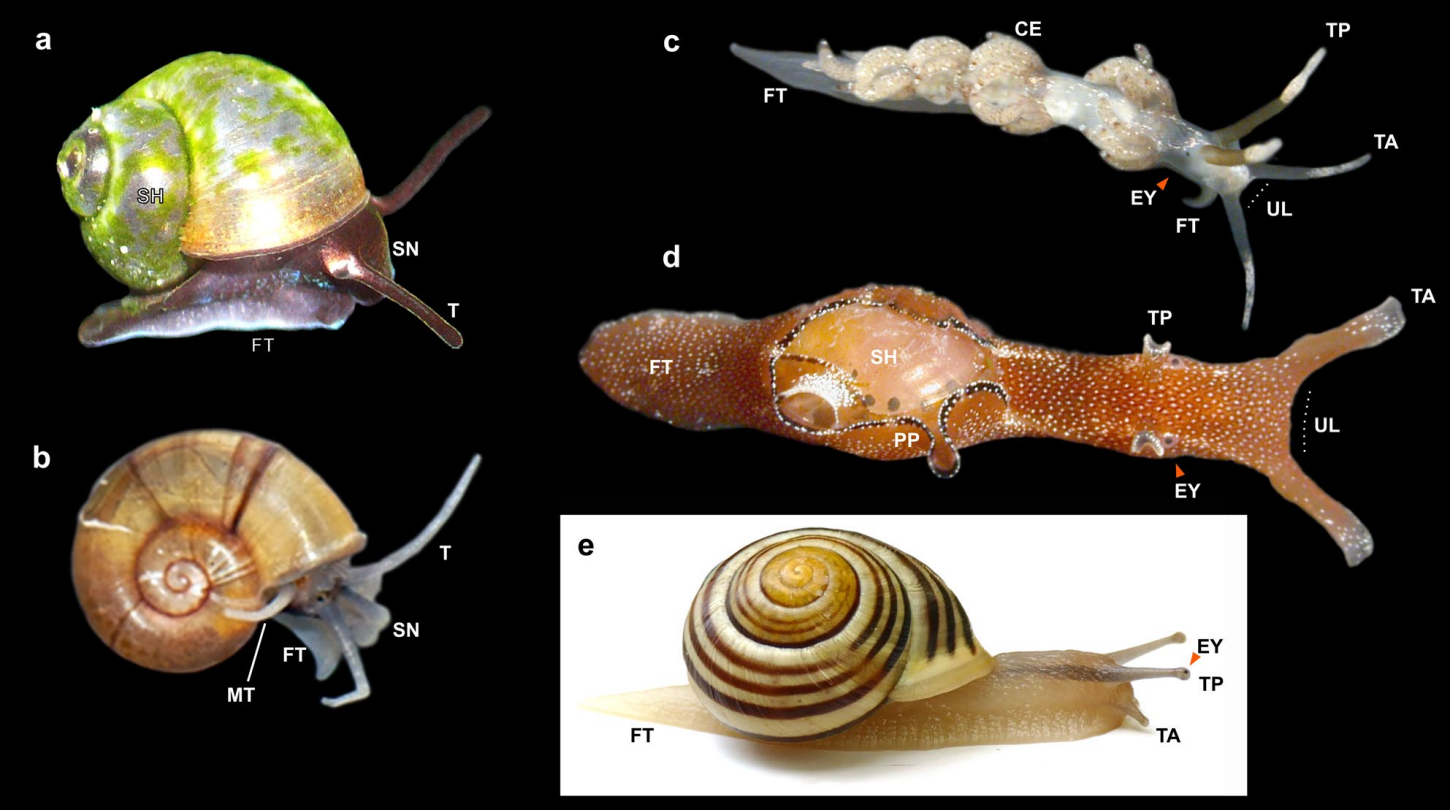
Comparative head-foot morphology of living Caenogastropoda and Heterobranchia. Macrophotographs of European species taken from above or right side. (**a**, **b**) Caenogastropoda and ‘lower’ Heterobranchia have one pair of smooth head tentacles and a short snout bearing the mouth at its tip. (**c–e**) Euthyneuran heterobranchs, in contrast, have one pair of anterior tentacles that form an upper lip above the mouth (labial/oral tentacles) and another separate pair of posterior tentacles (rhinophores or ommatophores). (**a**) Freshwater caenogastropod *Sadleriana bavarica* (2.2 mm in shell diameter; family Hydrobiidae). (**b**) Freshwater ‘lower’ heterobranch *Valvata cristata* (3 mm; Valvatidae); unpaired tentacle on right side is secondary and non-cephalic. (**c**) Marine nudibranch *Favorinus branchialis* (6 mm in body length; Favorinidae) with modified rhinophores; anterior foot extended to form tentacle-like projections. (**d**) Juvenile of marine euopisthobranch *Aplysia punctata* (10 mm; Aplysiidae) with two pairs of enrolled tentacles. (**e**) Terrestrial panpulmonate *Cepaea hortensis* (20 mm; Helicidae) with stalked eyes on posterior tentacles. Abbreviations: CE, cerata (dorsal appendages of mantle); EY, eye (highlighted by orange arrowheads); FT, foot; MT, unpaired tentacle extending from mantle margin; PP, parapodia (lateral extensions of foot covering shell); SH, shell; SN, snout; T, head tentacles; TA, anterior head tentacles; TP, posterior head tentacles; UL, upper lip or velum above mouth (highlighted by dotted lines).

A distinct part of molluscan biodiversity—about 40% of the estimated 73,000 described species^1^—is comprised by the long-recognized infraclass Euthyneura in the gastropod subclass Heterobranchia^2^. Euthyneurans play important and diverse ecological roles as predators, prey, or pests in various marine habitats (both benthic and pelagic), in freshwater realms, and on land^3,4^. Nudibranch sea slugs, sea hares, and garden snails with stalked eyes (Fig. 1c–e) are well-known euthyneurans that exemplify a fraction of derived morphologies found in this clade. Research has profited from their complex hermaphroditism (e.g.^5,6^) and sophisticated acquisition of defensive chemicals or ‘stolen’ cnidocytes and chloroplasts from their food (^7^ for review). Moreover, their so-called ‘giant’ neurons have enabled us to study individual nerve cells, neuronal circuits, and learning^8,9^.

The clade Euthyneura was named after its nervous system that lacks much of the asymmetry caused by gastropod torsion (twisting of the posterior body); ‘euthyneury’ or secondary detorsion was contrasted to ‘streptoneury’ with crossing posterior nerve fibres (^10^, Ponder et al. 2019: p. 340–346, 378–380^11^). Euthyneurans can more easily be distinguished from other gastropods by their head morphology in having two distinct pairs of head tentacles (with diverse shapes or reductions) formed by independent sets of anterior and posterior sensory areas (Fig. 1c–e;^12–14^). The lower, anterior tentacles (termed oral or labial tentacles) are specialized for contact chemoreception or ‘tasting,’ and fused in the middle to form the upper lip above the mouth. The upper, posterior tentacles (called rhinophores or eyestalks) are variable in shape but primarily responsible in distance chemoreception or ‘smelling’ (olfaction) and also the detection of water or air currents^3,12,14–17^.

**Figure 2:**
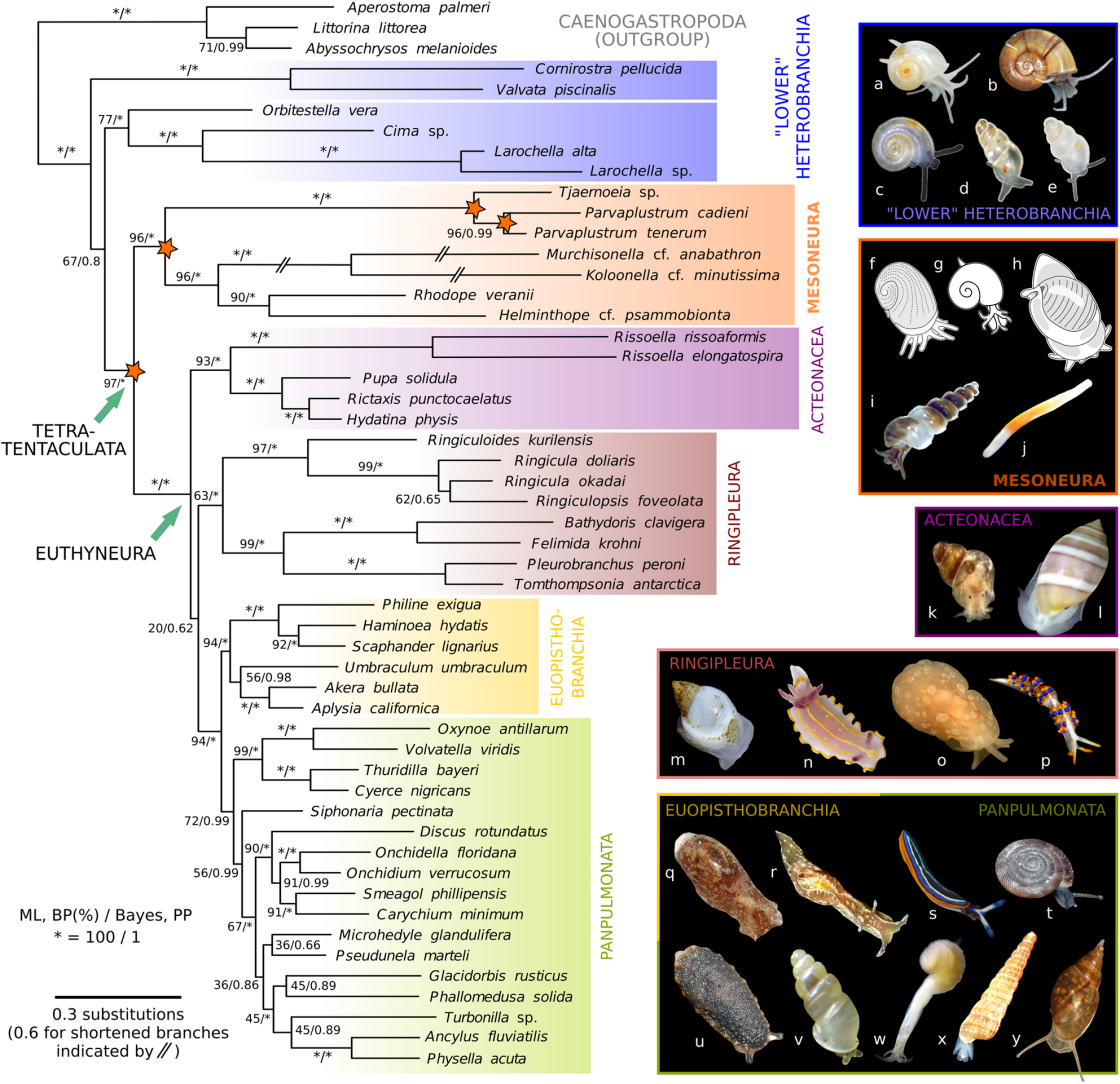
Molecular phylogeny of Heterobranchia and its new infraclass Mesoneura as sister to Euthyneura. Tree reconstruction was performed in MrBayes and based on combined nucleotide sequences of nuclear 18S and 28S rRNA and mitochondrial 16S rRNA and COI genes. Numerals on branches denote bootstrap proportions from RAxML-HPC analysis (BP in %, left) and Bayesian posterior probabilities (PP, right); asterisks denote full support (BP: 100%, PP: 1). Three branches of Murchisonellidae were shortened by 50% for graphical purpose. Lower heterobranch Architectonicidae and *Ammonicera* not included due to very long branches (see Supplementary Fig. S2a). Orange asterisks indicate four novel clades retrieved in this analysis; from left to right: Tetratentaculata (no rank), new infraclass Mesoneura, new superfamily Tjaernoeioidea, and new family Parvaplustridae. (**a–y**) Exemplar species from major subgroups of Heterobranchia: (**a**) *Tomura yashima* (representing Cornirostridae), (**b**) *Valvata cristata*, (**c**) *Boschitestella* cf. *eloiseae* (Orbitestellidae), (**d**) *Cima* sp., (**e**) *Larochella* sp., (**f**) *Tjaernoeia exquisita* (redrawn and modified from Jensen, 1999: fig. 2A^26^), (**g**) *Tj. exquisita* (redrawn and modified from Warén, 1991: fig. 26^27^), (**h**) *Parvaplustrum tenerum* (redrawn and modified from Powell, 1951: fig. 97^28^), (**i**) *Murchisonella* cf. *anabathron* (photograph courtesy of Angela Dinapoli), (**j**) *Rhodope veranii*, (**k**) *Rissoella diaphana*, (**l**) *Acteon tornatilis*, (**m**) *Ringicula doliaris*, (**n**) *Felimida krohni*, (**o**) *Berthella ocellata* (Pleurobranchidae)*,* (**p**) *Trinchesia morrowae* (Nudibranchia: Trinchesiidae), (**q**) *Haminoea hydatis*, (**r**) *Aplysia punctata*, (**s**) *Thuridilla hopei*, (**t**) *Discus rotundatus*, (**u**) *Onchidella celtica*, (**v**) *Carychium pessimum*, (**w**) *Pseudunela marteli*, (**x**) *Turbonilla rufa*, and (**y**) *Physella acuta*.

In contrast, the other Gastropoda, namely the paraphyletic ‘prosobranchs’ (including the grade of the so-called ‘lower’ Heterobranchia; Fig. 1a,b), generally are streptoneurous. They usually have no horizontal upper lip but have a short, tube-shaped snout and only one pair of head tentacles which is, curiously, often innervated by a deeply forked nerve that sends two parallel branches into each tentacle (e.g.^18^, but see^19,20^ for discussion). Individual function of these parallel nerve branches has not been explored, but the latest phylogenetic analyses of Gastropoda^21,22^ confirm that this forked nerve is ancestral for Heterobranchia and its sister-group Caenogastropoda (collectively referred to as Apogastropoda^19,20,23^) (Figs. 1b, 2a–e). Some previous authors have suggested homology of the prosobranch (and thus lower heterobranch) head tentacle to only the labial tentacle of Euthyneura (see e.g.^24,25^). More recently, Staubach (e.g. fig. 32)^13^, based on detailed neuro-anatomical data for many taxa, hypothesized homology of the single tentacle pair of prosobranchs to both of the specialized euthyneuran tentacles (see also^14,20^).

Combined morphological and phylogenetic research has continuously refined the understanding of heterobranch and euthyneuran evolution^29–35^. Phylogenetic methods today have the potential to robustly reconstruct backbone trees of biological relationships even for ancient evolutionary events (see^36,37^), which in turn help to trace pathways of evolutionary changes on both large and fine scales. The inclusion of minute and difficult-to-collect taxa have further enhanced resolution and interpretative power of such approaches (e.g.^33,38,39^). Within the larger framework of reconstructing the early evolution of heterobranch gastropods, we here conducted a phylogenetic analysis using multi-locus molecular markers that are well-established for the study of the group^33,35,40^. We here added for the first time members of the enigmatic, primarily deep-water genera *Tjaernoeia* Warén & Bouchet, 1988 and *Parvaplustrum* Powell, 1951 (Fig. 2).

*Tjaernoeia* species are among the smallest living gastropods (< 1 mm). They have been classified in their own family Tjaernoeiidae Warén, 1991, and tentatively among the Euthyneura^2^. Five described species from the Atlantic and Antarctica have coiled shells with a characteristic dimpled surface^26,27^. The three described species of *Parvaplustrum* have been classified as dubious members of the euthyneuran clade Acteonoidea based on their fragile, oval ‘bubble’ shells and presence of an external copulatory organ^28,41,42^. Interestingly, although not commented upon by previous authors^26,27,42,43^, *Tjaernoeia* and *Parvaplustrum* principally resemble lower heterobranchs in the outline of head-foot morphology but also share a unique condition of long, basally forked head tentacles. We found that inclusion of these taxa in a phylogenetic analysis greatly improves resolution of relationships within Heterobranchia and provides a novel scenario on the evolution of the head tentacles and nervous system of Euthyneura.

## Results and discussion

### Mesoneura, a new clade sister to euthyneuran Heterobranchia

**Figure 3:**
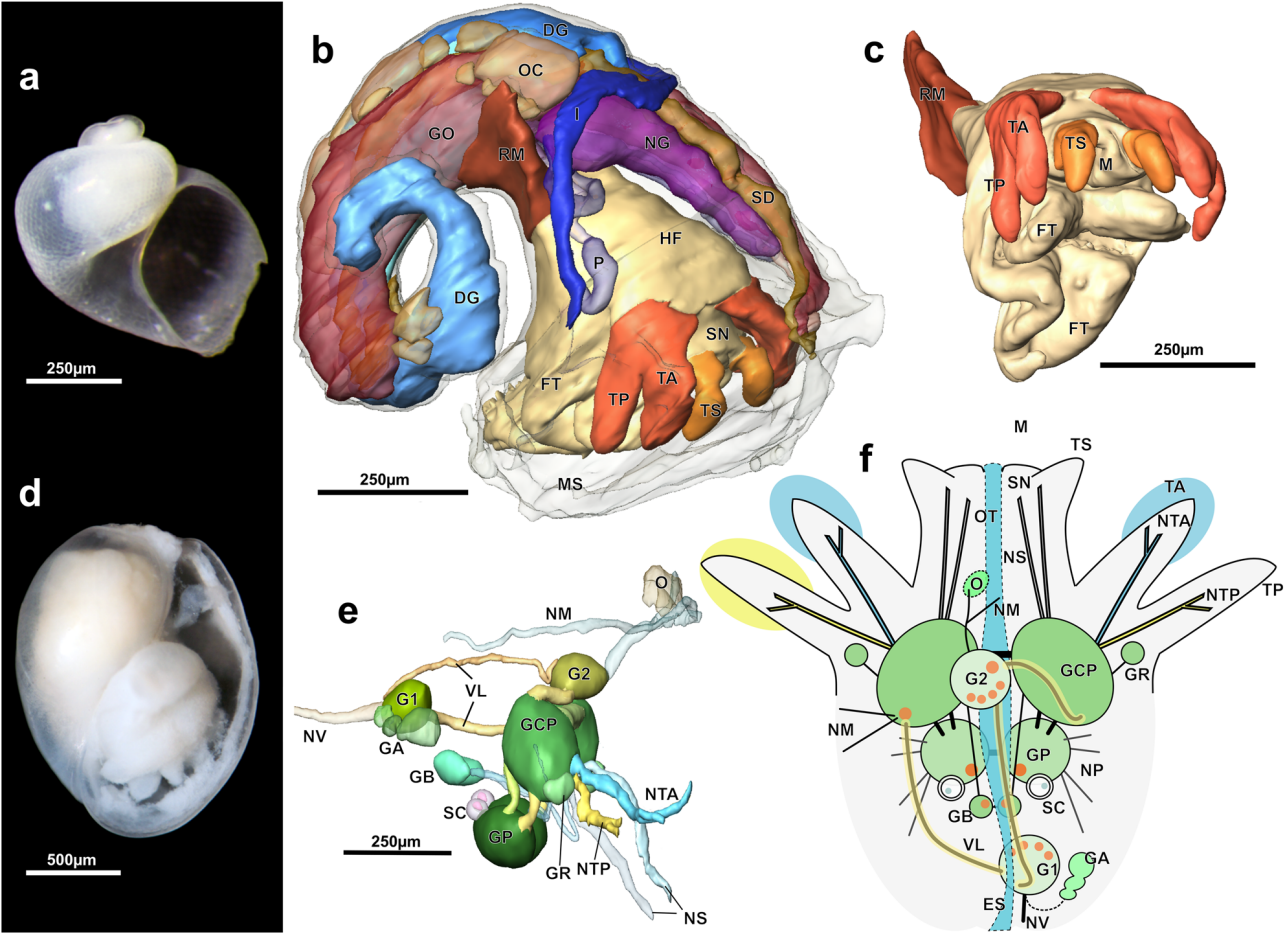
Micromorphology of superfamily Tjaernoeioidea nov. with focus on head tentacles and central nervous system. (**a–c**) Species of genus *Tjaernoeia* Warén & Bouchet, 1988. (**a**) Undescribed *Tjaernoeia* sp. from off Aomori, Japan (sequenced specimen, AORI YK#2783). Apertural view of shell, showing a dimpled surface and a slightly sinistral apex. (**b**) European *Tjaernoeia exquisita,* 3D reconstruction of preserved animal (ZSM Mol-20200024), right anterodorsal view. Shell removed, outer skin of mantle shown in transparency, organs of mantle roof omitted for clarity. (**c**) Same as b, anteroventral view, showing detail of head-foot with bifid tentacles (red). (**d–f**) South Atlantic *Parvaplustrum tenerum* Powell, 1951. (**d**) Apertural view of shell and preserved soft body inside (voucher specimen ZSM Mol-20020851); shell ‘bubble-shaped’ with a sunken apex at top. (**e**) 3D-reconstruction of central nervous system, right view (ZSM Mol-20021303). Cerebral nerves of left side (NTA, NTP, NS) omitted for clarity. (**f**) Schematic drawing of head and central nervous system showing innervation of bifid tentacles, dorsal view, anterior side up. Orange circles indicate positions of histologically distinct neurons, which are 3–4 times larger in diameter than other neurons and potential homologue of ‘giant’ neurons in *Aplysia*^8^. Abbreviations: DG, digestive gland; ES, oesophagus; FT, foot; GA, cluster of accessory ganglia near copulatory organ; GB, buccal ganglion; GCP, cerebropleural ganglion; GO, gonad; GP, pedal ganglion; GR, accessory (rhinophoral?) ganglion; G1, posteroventral visceral ganglion; G2, anterodorsal visceral ganglion ; HF, head-foot; I, intestine; M, mouth; MS, mantle skirt; NG, nidamental gland; NM, nerves to mantle; NS, nerves to snout; NTA, nerve of anterior tentacle; NTP, nerve of posterior tentacle; NP, nerves to foot (4 pairs); NV, nerve to visceral sac (two only on left side); O, sensory osphradium with ganglion; OC, mature oocytes; OT, oral tube; P, penis; RM, retractor muscle; SC, statocyst with single statolith; SD, seminal duct; TA, anterior head tentacle; TS, lateral projection on snout; TP, posterior head tentacle; VL, slightly streptoneurous visceral loop.

Our molecular phylogenetic reconstruction (Fig. 2, Supplementary Fig. S1; Table 1) recovered *Tjaernoeia* sp. from off Japan (Fig. 3a), *Parvaplustrum tenerum* Powell, 1951 from the South Atlantic and *P. cadieni* Valdés, Gosliner & Warén, 2017 from off California as a maximally supported clade in both Maximum-Likelihood (ML) and Bayesian analyses (bootstrap proportion, BP: 100%, transfer bootstrap expectation, TBE: 100%, Bayesian posterior probability, PP: 1). With this topology, we reclassify the genus *Parvaplustrum* into a new monotypic family of bubble snails, Parvaplustridae nov., which is sister to Tjaernoeiidae. A new superfamily Tjaernoeioidea is also erected to contain the families Tjaernoeiidae and Parvaplustridae nov. Surprisingly, this new superfamily formed a strongly supported sister (BP: 96%, TBE: 93%, PP: 1; Fig. 2f–h) to the recently recognized Allomorpha, which contains morphologically divergent rhodopid slugs and murchisonellid snails^39^. Allomorpha was confirmed in the present study as a monophyletic group with strong support (96%, 98%, 1; Fig. 2i, j). The clade Tjaernoeioidea nov. + Allomorpha, here named as a new infraclass Mesoneura, was identified as a robustly supported sister to the over 30,000 species of the infraclass Euthyneura (97%, 96%, 1; Fig. 2k–y). We here propose the name Tetratentaculata nov. for the clade of Mesoneura nov. and Euthyneura. Mesoneura presents a novel taxon in a phylogenetic position between the species-rich clade Euthyneura and a less-diverse grade of ‘lower’ heterobranchs (Fig. 2a–e).

**Table 1:** Summary of sequence alignment and phylogenetic signals for individual genes and concatenated datasets. Bootstrap proportion (BP) and transfer bootstrap expectation (TBE) are shown as percentages for each clade; bold letters denote significant support (≥ 80%); N, not recovered in best ML tree; NA, not applicable. Sensitivity analyses were run with three additional lower heterobranchs (w/AA) or without Rhodopidae (wo/Rho) or Murchisonellidae (wo/Mur). See Supplementary Figs. S1 and S2.

	Nuc rRNA			MtDNA			All (4 genes)	Sensitivity analyses		
	18S	28S	(18S + 28S)	16S	COI	(16S + COI)		w/AA	wo/Rho	wo/Mur
Number of taxa	47	50	51	50	49	51	**52**	55	50	50
Total length (MAFFT)	2474	1640	4114	522	657	1179	**5293**	5787	5216	5232
Final alignment (Gblocks)	1689	947	2636	336	657	993	**3629**	3618	3644	3628
Variable sites	620	594	1214	223	417	640	**1854**	2025	1851	1799
Parsimony informative	417	497	914	209	377	586	**1500**	1740	1497	1464
Clade, BP/TBE (%)										
** Tjaernoeioidea nov**	**100/100**	**100/100**	**100/100**	**97/94**	**100/100**	**98/90**	**100/100**	**100/100**	**100/100**	**100/100**
Rhodopidae	**93/93**	**94/87**	**84/86**	**92/82**	(NA)	74/59	**90/87**	**94/93**	(NA)	**100/100**
Murchisonellidae	(NA)	**100/100**	**100/100**	**96/81**	N/N	**93/90**	**100/100**	**100/100**	**100/100**	(NA)
Allomorpha	(NA)	74/77	68/79	N/N	N/**88**	48/N	**96/98**	**88/96**	(NA)	(NA)
** Mesoneura nov**	N/N	69/74	54/72	N/N	77/**93**	N/73	**96/93**	64/**83**	**83/93**	78/64
Euthyneura	N/N	36/69	40/74	N/N	**95/99**	**93/99**	**100/100**	**95/98**	**91/95**	**97/98**
** Tetratentaculata nov**	N/N	N/N	36/62	N/N	53/**84**	N/74	**97/96**	49/74	**89/92**	63/**81**

The present tree topology otherwise conformed to previous studies using either comparable marker and taxon sets^33,35,40^, mitogenomics^44,45^, or phylogenomics^21,46^ in retrieving the paraphyletic ‘lower’ heterobranchs on the one hand and the monophyletic Acteonacea (or Acteonimorpha), Ringipleura, Euopisthobranchia and Panpulmonata as constituting Euthyneura on the other (some previous authors classified Acteonacea as lower heterobranchs, e.g.^33^). Internal support values of derived clades (i.e., euopisthobranchs and panpulmonates) were lower than in comparable studies with more expansive taxon sampling of these groups^33,47^. Interestingly, careful BLAST searches for the present and previous Genbank sequences, followed by the removal of ambiguous or misidentified data (see Supplementary Table S2), resolved the topology of the lower heterobranchs differently from previous studies (Fig. 2a–e;^39,40^): Orbitestellidae with discoidal shells were recovered as the sister (77%, 87%, 1) to high-spired Cimidae (*Cima* and *Larochella*; full support), instead of having these taxa separate in a large grade^40^ or in a polytomy^39^.

This topology was stable to sensitivity analyses where taxa were selectively added or removed (Table 1, Supplementary Fig. S2). Inclusion of the only remaining lower heterobranch clades Architectonicoidea (presumed here to encompass Mathildidae based on morphology) and *Ammonicera* (currently classified as ‘Omalogyroidea’) into separate ML analyses found these to form a monophyletic taxon (BP: 100%, TBE: 100%) as sister to Valvatoidea but with low support (45%, 72%) and an extremely long branch (Supplementary Fig. S2). Inclusion of this long-branched clade resulted in the masking of more numerous alignment-ambiguous sites and thereby lowered nodal support over the tree, yet without significantly affecting the topology. Exclusion of either Rhodopidae or Murchisonellidae also resulted in the same topology with the monophyletic Mesoneura (BP: 78–83%, TBE: 64–93%) and Tetratentaculata (63–89%, 81–92%) (Table 1; Supplementary Fig. S2).

### Comparative microanatomy of Tjaernoeioidea

Past morphological studies on *Tjaernoeia* and *Parvaplustrum* were confounded by their extremely small body sizes, which resulted in the examination of only the shell, external anatomy and chitinous internal structures (^26–28,41–43^). Species of *Tjaernoeia* bear a broad, somewhat flattened, coiled shell, similar to those of some other heterobranchs, in particular lower heterobranch taxa such as the Valvatoidea^27^. *Parvaplustrum* species on the other hand have a fragile, oval and inflated ‘bubble’ shell with a sunken spire and without an umbilicus, resembling those of many euthyneuran groups (e.g. *Hydatina* and *Haminoea*, see Fig. 2q)^28,41–43^. Based on our study of aligned serial histological sections and three-dimensional reconstructions of the body shape and internal organs, we here show aspects of microanatomy for the type species of each genus, namely the North Atlantic *Tjaernoeia exquisita* (Jeffreys, 1883)(Fig. 3b,c) and South Atlantic *P. tenerum* (Fig. 3d–f). Particular focus is here placed on the head morphology and configuration of the central nervous system (CNS) in comparison with other gastropods including newly studied *Ebala* (Mesoneura: Allomorpha: Murchisonellidae) and *Rissoella* (Euthyneura: Rissoellidae) (see Supplementary Fig. S3). The CNS is of particular interest due to its conservativeness during gastropod evolution, with neuronal connections inside its ganglia conserved for hundreds of millions of years^13,14^.

The herein reconstructed, preserved animal of *T. exquisita* was reproductively mature, yet only 550-µm long (Fig. 3b) and much smaller than *P. tenerum* (ca. 2 mm; Fig. 3d). Although with different sizes and shell shapes, the two species are very similar to each other in head-foot morphology. They both resemble lower heterobranchs including the Cimidae and many of Valvatoidea (see e.g. Fig. 2a,b,d) in having a slender and anteriorly bifurcated foot and a short snout^27^. This snout bears a short, finger-shaped tentacle on either side of the tip (TS), as seen in some species of Valvatoidea^18^. More posteriorly, the head of *T. exquisita* and *P. tenerum* has two conspicuous pairs of long, deeply bifurcated and slightly flattened head tentacles of which the anterior branch is about 30–40% shorter than the posterior one (TA and TP in Fig. 3b,c,f). The surface of these tentacles is uniformly smooth, with interspersed gland cells and with ciliation mainly on the inner side. Eyes are lacking in both species, and the body is colourless (see also^27^). The posterior side of the foot lacks an operculum in both *Tjaernoeia* and *Parvaplustrum*, as in the Rhodopidae and the majority of Euthyneura, whereas the Murchisonellidae and lower heterobranchs are all operculate^48,49^.

The general similarity of their head-foot is also reflected in the nervous system of *Tjaernoeia* and *Parvaplustrum*. The CNS, shown here in the larger-bodied *P. tenerum* (Fig. 3e,f), contains a cerebral nerve ring with four ganglia (paired cerebropleural and pedal ganglia: GCP and GP) and two buccal ganglia (GB) below the pharynx. The asymmetric, twisted visceral nerve loop (VL) encircles the oesophagus and bears dorsal (G2) and posteroventral ganglia (G1). *Parvaplustrum tenerum* differs from *T. exquisita* in having small additional ganglia of unknown function joined to the sides of the cerebropleural ganglia (GR). Moreover, only *P. tenerum* has a cluster of accessory ganglia (GA) at the base of the external copulatory organ (P) on the right side of the head behind the bifid tentacles; this copulatory organ in *P. tenerum* is larger than that of *T. exquisita* and bears a chitinous stylet (not shown). In both species, several paired nerves emanate from the cerebropleural ganglia. The snout is innervated by two pairs (in *P. tenerum*) or a single pair (in *T. exquisita*) of nerves. The bifurcated head tentacles are each innervated by two independent nerves that emerge directly adjacent to each other (NTA, NTP); these nerves do not bear obvious lateral ramifications except two small branches near the nerve tip in *Parvaplustrum*.

In addition, certain ganglia of *P. tenerum* (GP, GB, G1, G2 and left GCP) have several large neurons (orange circles in Fig. 3f) that are histologically and topologically identifiable with the ‘giant’ neurons of the neurobiological model organism *Aplysia* (Figs. 1d, 2f) and many other euthyneurans (Gillette, 1991: p. 235^8^). These cells could not be identified in *T. exquisita* with its smaller body size, as was previously the case in Rhodopidae^48^ and Murchisonellidae^49^. The two species resemble lower heterobranchs in lacking specialized epidermal sensory areas innervated by smaller branches of the tentacle nerves, such as the so-called Hancock’s organs (which are present at the base of posterior head tentacles of most aquatic euthyneurans;^14^; see below). The osphradium, another chemosensory organ in the molluscan pallial cavity, is present in both *T. exquisita* and *P. tenerum* as a small ciliated patch of epidermis on the anterior roof of the mantle (O in Fig. 3e,f).

The different shell shapes of *T. exquisita* and *P. tenerum* are also reflected in the different organization of their mantle. Specifically, *P. tenerum* has its plicate gill, glands and kidney all located on the posterior right of the mantle (a condition called ‘detorted’ in Euthyneura). On the other hand, in *T. exquisita* the gill is a more medially lying, microscopic leaf without folds, glands are spread along the anterior margin of the mantle, and the kidney lies centrally, reflecting a more ancestral condition typical of non-euthyneuran heterobranchs (e.g.^10,18^). Opposing ciliary strips in the mantle cavity, regarded as an apomorphy of Heterobranchia^10,29^, could not be reliably identified in *Tjaernoeia* and *Parvaplustrum* and among the Mesoneura such structures are so far only confirmed for some Murchisonellidae^50^. *Tjaernoeia* and *Parvaplustrum* are similar in their complex hermaphroditic reproductive system (Fig. 3b) and in the simple digestive tract with a very narrow cuticular radula that was shown by other authors to bear only leaf-shaped lateral teeth^27,41,43^.

Morphological diagnoses of the newly proposed taxa can be summarized as follows: **Tjaernoeioidea**, a new superfamily for *Tjaernoeia* (Tjaernoeiidae) and *Parvaplustrum* (Parvaplustridae nov.): Small to minute heterobranch snails with a single pair of deeply bifid head tentacles (or, depending on perspective, two pairs of basally joined tentacles) and a slender snout with a lateral projection on either side; eyes lacking and skin unpigmented; external copulatory organ on the right side of the head-foot; shell fragile, globose to oval, smooth or with sculpture of small dimples, protoconch hyperstrophic; foot without an operculum. **Parvaplustridae**, a new monotypic family for *Parvaplustrum* [ZooBank registration (LSID): urn:lsid:zoobank.org:act:A7416D49-113A-4E60-86A7-6AE46818B20A]: Shell inflated, oval, with a large aperture and an involute spire; shell surface smooth or with minute, irregularly scattered pits; mantle detorted with the gill, kidney and large glands all located on the posterior right; copulatory organ with a tubular chitinous stylet. We regard these differences in external morphology to warrant separate family status. **Mesoneura**, a new infraclass for Tjaernoeioidea + Allomorpha: Named after its nervous system showing a mix of plesiomorphic and apomorphic conditions in their nervous system, with tentacle nerves that innervate independent areas of the head but otherwise largely lack ramifications; Hancock’s organ and median lip absent; visceral loop with torsion yet long; radula (if present) narrow without a rachidian tooth and with only one slender lateral tooth on either side of a transverse row. **Tetratentaculata**, a new clade name for Mesoneura + Euthyneura: Head with four individual sensory areas corresponding to four tentacles or two deeply bifurcated tentacles, although tentacles per se may be reduced secondarily; giant neurons present in the pedal, buccal, dorsal, posteroventral, and left cerebropleural ganglia (see below); visceral nerve cord at least slightly detorted or completely detorted. Accordingly, Euthyneura can be newly diagnosed as tetratentaculate heterobranchs distinguished from Mesoneura by having (1) labial tentacles that are medially fused to form an upper lip or velum and (2) tentacle nerves bearing many small ramifications that innervate sensory cells including those of the Hancock’s organ (see discussion below).

### Origin of euthyneuran head tentacles

The herein recovered phylogenetic position of the Tjaernoeioidea corroborates a recent morphology-based hypothesis^13,20^ that the two pairs of specialized euthyneuran head tentacles might have originated through bifurcation of an ancestral single pair of tentacles, where each tentacle was already innervated by two nerve cords, as now seen in the Caenogastropoda and lower Heterobranchia^3^. It was previously assumed that only the anterior pair (labial tentacles) of the Euthyneura was homologous to the plesiomorphic head tentacles of the Gastropoda (see^13,24,51^). The posterior pair (rhinophores or ommatophores or eyestalks) was regarded as secondarily acquired or even repeatedly acquired (e.g.^25^). However, convincing evidence for the homology of rhinophores across the Euthyneura came from the use of axonal backfilling techniques^12,14,15^ that reliably identified and correlated individual tentacle nerves across a broad set of taxa. Furthermore, Staubach^13^ identified highly conserved neuron clusters associated with the tentacle nerves in the cerebral ganglia of the periwinkle (Caenogastropoda: *Littorina*) and giant African snail (Heterobranchia: Stylommatophora: *Achatina*). This conservatism in details of neuronal architecture of tentacle innervation further supports homology of tentacles across the Apogastropoda (see Fig. 1, Supplementary Fig. S3).

**Figure 4:**
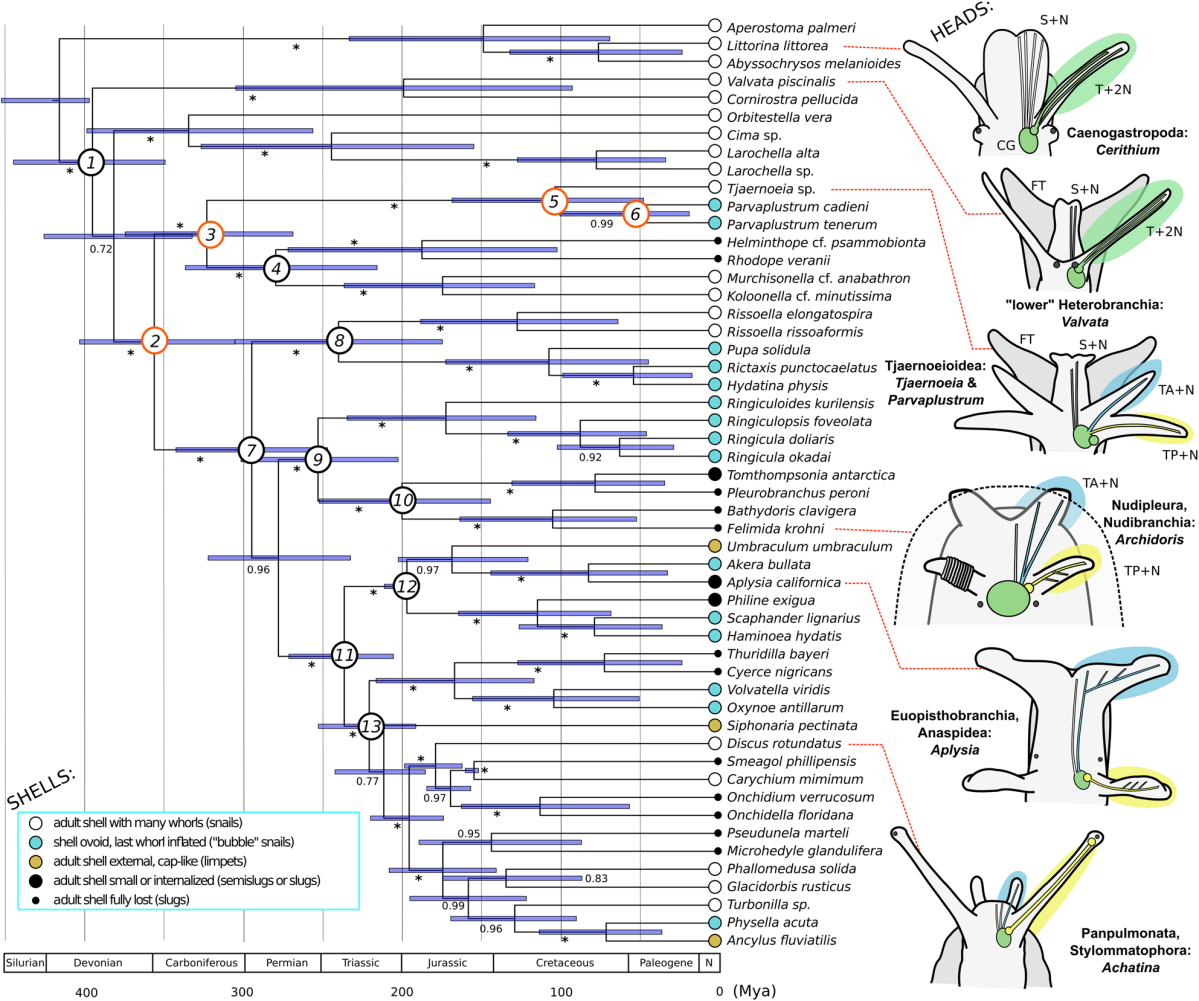
Time-calibrated phylogeny of heterobranch gastropods and distributions of different types of head and shell morphologies. Tree reconstruction was based on combined four-gene sequences and calibration priors placed on three nodes and performed in BEAST. Numerals on branches are Bayesian posterior probabilities; asterisks denote full support (PP: 1). Numbered circles at nodes indicate major clades, including (1) Heterobranchia, (2) Tetratentaculata nov., (3) Mesoneura nov., (4) Allomorpha, (5) Tjaernoeioidea nov., (6) Parvaplustridae nov., (7) Euthyneura*,* (8) Acteonimorpha, (9) Ringipleura, (10) Nudipleura, (11) Tectipleura, (12) Euopisthobranchia, and (13) Panpulmonata. Pictograms of head morphology suggest two pairs of tentacles existed at node 2 (hence new name Tetratentaculata: ‘bearing four tentacles’). Colour-coded circles at terminals show phenotypic plasticity of shells in Tetratentaculata, particularly in Euthyneura. Abbreviations: CG, cerebral/cerebropleural ganglion; FT, foot; S + N, snout with snout nerves; T + 2N, single tentacle with two nerve cords (green area); TA + N, anterior/labial tentacle with one or two nerve pairs (blue area); TP + N, posterior tentacle or rhinophore/ommatophore with single nerve (yellow area). Head schemes of *Aplysia, Archidoris* and *Achatina* after^13^, anterior side is up. See Supplementary Fig. S3 for further comparison.

In combination with these results, our new findings lead to an evolutionary scenario of two steps. First, at the origin of Tetratentaculata, the single ancestral tentacle with double nerves split into two tentacles, each with one of the two ancestral nerve cords. Second, at the origin of Euthyneura, the two tentacles became specialized into the anterior and posterior tentacles, with different shapes and with more elaborate sensory areas such as the rhinophores and Hancock’s organs. The first of these evolutionary steps might still be visible in the extant Tjaernoeioidea, which have a pair of deeply bifurcated yet basally joined tentacles. With only few exceptions, the two pairs of ramified tentacle nerves—acquired in the second step—persist across the Euthyneura including groups with atypical tentacles (Fig. 4, Supplementary Fig. S3, see^3,11,13,14,24,35^).

The Allomorpha, although being sister to the Tjaernoeioidea, do not share the long and bifid head tentacles and thus might question the evolutionary scenario proposed above. However, allomorph snails and slugs may have modified or lost the bifid tentacles in relation to their sediment-dwelling or even infaunal lifestyles^39,48,49^ as have many euthyneurans^12,13,29,35^. Murchisonellids bear a pair of posterior, oftentimes broad, tentacles that are innervated with two pairs of essentially unbranched nerve cords (see^49^, and Supplementary Fig. S3 for reexamination of *Ebala*). Rhodopids have entirely lost the head tentacles per se but there remain the two pairs of the tentacle nerves in the head^39,48^. After a confused taxonomic history (see^39^), the Allomorpha are now found to resemble their previously unrecognized sistergroup Tjaernoeioidea more than other heterobranchs in having four separate tentacle nerves, but lacking unequivocal Hancock’s organs, distal ramifications of tentacle nerves, and a typical medially-fused upper lip^49^. Absence of the Hancock’s organ and upper lip also distinguishes the Tjaernoeioidea from the shallow-water, herbivorous euthyneuran snails of the superfamily Rissoelloidea (Supplementary Fig. S3), regardless of the short, bifid head tentacles of *Rissoella* that externally might recall those of tjaernoeioids (Fig. 2k;^52,53^).

### Shell shapes and radiation of Tetratentaculata over geologic time

Our BEAST analysis with the ages of three fossil heterobranchs as calibration points (Fig. 4) estimated that Tetratentaculata (node 2) originated sometime in the Devonian–Carboniferous period with a posterior mean age of 359 million years ago (Mya) and 95% highest posterior density (HPD) intervals of 406–307. The split between Tjaernoeioidea and Allomorpha (node 3; mean: 325 Mya, 95% HPD: 377–270) potentially predated any other split within the Tetratentaculata, whereas *Tjaernoeia* and *Parvaplustrum* appear to have diverged much more recently (node 5; 103 Mya, 169–47). The rhodopid stemline may extend into the late Palaeozoic (node 4; 281 Mya, 339–217), further back in time than other extant lineages of slugs (Fig. 4: black circles at branch terminals), save potentially the Nudipleura (node 9; 254 Mya, 303–203) or the Nudibranchia (node 10; 201 Mya, 253–144).

The speciose radiation of Euthyneura (node 7), leading also to diverse shell-shapes and instances of shell loss (Fig. 4: circles at branch terminals), was estimated to have started in the Carboniferous–Permian time (296 Mya, 345–248). This predates the oldest known euthyneuran fossils, the diverse Cylindrobullinoidea occurring since the Early Triassic (245 Mya) (see^54,55^ for discussion). Cylindrobullinoids have been suspected to contain paraphyletic or polyphyletic members of already diverged euthyneuran lineages (see^55^ for review), some of which may lead to the extant Acteonimorpha, Ringipleura, Euopisthobranchia and Panpulmonata. The early Euthyneura are suggested to have a characteristic bubble shell with a large body whorl—a morphology found in several lineages of the extant Euthyneura (Fig. 4: blue circles at terminals)—as well as a hypertrophied foot and headshield for an oftentimes infaunal mode of life^35^. The morphological variability found in Mesoneura does not allow unequivocal reconstruction, but they display bubble-shell and slug morphotypes (Parvaplustridae and Rhodopidae) in parallel with the Euthyneura. Fossils of putatively ancestral murchisonellids (as Donaldinidae and Streptacididae, see^56^) come from the strata of 350–260 Mya, which is much older than the first occurrences of the fossil Cylindrobullinoidea and closely fit the herein proposed age of divergence (node 3 in Fig. 4, see above). Some of those Palaeozoic taxa^57–59^are indeed fairly similar to the modern *Murchisonella*^60,61^ in teleoconch and protoconch morphology. On the other hand, certain early-Triassic fossils have a murchisonellid-like protoconch and a cylindrobullinoid-like teleoconch and were therefore interpreted as a phylogenetic link between the Streptacididae and Cylindrobullinoidea^56^.

The Euthyneura comprise almost half of all molluscan species richness —why could they have radiated into so many ecological niches and diversified into so many species? Kano et al.^35^ hypothesized that early euthyneurans were freed from the strict connection of the shell and mantle margin, thereby releasing the mantle from morphological constraints and allowing the creation of evolutionary novelty. We here add that the modification of the head, although not evident in the fossil record, seems to present another overlooked key event in their evolutionary history. The diverse nature of head tentacles is considered important for the taxonomy of euthyneuran subgroups, particularly those of sea slugs^14,25,34^. However, understanding of their role in euthyneuran evolution as a whole has been overshadowed by a focus on shell loss and coinciding chemical defence. The diverse rhinophores, eyestalks, and Hancock’s organs of Euthyneura play crucial roles in directional chemosensing^11,13,14,25,62^ and are much more specialized than head tentacles in other gastropods (shown in Fig. 4 at right; Supplementary Fig. S3). For the other (aquatic) gastropods, the osphradium in the mantle cavity is the primary chemosensory organ^3^, but it is generally simplified in euthyneuran and non-euthyneuran heterobranchs^63,64^. This suggests that, at least in Euthyneura, sensory capacity of the head has not only become more pronounced relative to other Gastropoda but it has also functionally replaced much of mantle-based chemosensing, as implied by previous authors (see Morton, 1972: p. 337^65^; Gosliner, 1994: p. 346^25^). We here suggest that the acquisition of the more elaborate head sensors has determined the observed shift of euthyneuran ecology towards more motile and predatory lifestyles, often with specialized prey items and habitats^66,67^. Furthermore, reduced reliance on the osphradium for chemosensing may have removed constraints to reorganization of the mantle, allowing additional evolutionary plasticity and innovations in the morphology of the posterior body and shell. The acquisition of the enhanced head sensors may therefore be linked to the shell loss and also to the explosive radiation and speciation of Euthyneura.

This study highlights that inclusion of rare, microscopic taxa in an integrated analysis has the potential to greatly improve the resolution of phylogenetic relationships and provides a novel scenario on a large-scale evolutionary process, in the present case within heterobranch and euthyneuran gastropods.

## Methods

### Sampling and preparation of specimens

Living snails of the following seven heterobranch species were collected from coastal to bathyal waters using various methods (see below; Supplementary Tables S1, S2 and Supplementary Fig. S3 for additional data). Specimens were preserved either (1) directly in 95–99% ethanol, or (2) fixed in a 10% formalin-seawater solution after anesthetization in isotonic magnesium chloride solution and then transferred to 80% ethanol. All sectioned specimens and DNA extracts are deposited at the Mollusca section of the Bavarian State Collection of Zoology, Munich, Germany (ZSM) or at the Atmosphere and Ocean Research Institute, The University of Tokyo, Kashiwa, Japan (AORI).

*Parvaplustrum tenerum* Powell, 1951 [museum voucher numbers ZSM Mol-20021303/2 (DNA aliquot B103), ZSM Mol-20020851 (embedded specimen block 1W7), ZSM Mol-20021303/1 (block 2W5, 3D-reconstructed)]: Collected in 2002 at Burdwood Bank, SW of Falkland Islands, South Atlantic (272 m, 54°02’S, 62°02’W, Agassiz Trawl) during LAMPOS expedition, R/V Polarstern cruise ANTXIX-5, station PS61/145–1. Vouchers preserved in 96% ethanol; DNA extracted and used for phylogenetic analysis (Figs. 2, 4) or embedded in Epon epoxy resin and serially sectioned, used for 3D reconstruction (Fig. 3d–f).

*Parvaplustrum cadieni* Valdés, Gosliner & Warén, 2017 [Swedish Museum of Natural History SMNH-111919 (tissue clip AORI YK#2783)]: Collected in 2010 at Hydrate Ridge, off Oregon, USA (795 m, 44° 34′N, 125° 09′W) during R/V Atlantis (AGOR-25) cruise AT15-68, DSV ALVIN dive 4635, by Anders Warén. Voucher preserved in pure ethanol, clipped tissue used for phylogenetic analysis (Figs. 2, 4).

*Tjaernoeia exquisita* (Jeffreys, 1883) [ZSM Mol-20200024 (block 33B)]: Collection date and details unknown, presumably before 2000 in Skagerrak, Sweden by subtidal dredging, collected and prepared by the late Cristoffer Schander. Voucher fixed and preserved in Formalin (?), embedded in Araldite epoxy resin and serially sectioned, 3D-reconstructed (Fig. 3b,c). [Note that Appolloni et al.^68^ consider *Tj. imperspicua* (Monterosato, 1875) (non Chaster, 1895) to be the oldest valid name for this species, although it is generally regarded a *nomen nudum*^27^].

*Tjaernoeia* sp. (AORI YK#2783): Collected in 2012 from off Hachinohe, Aomori Prefecture, Honshu, Japan (459–498 m, 40°58’N, 141°46’E) during R/V Tansei-maru cruise KT-12-18, by Yasunori Kano. Voucher preserved in 99% ethanol, clipped tissue used for phylogenetic analysis (Figs. 2, 4), shell imaged (Fig. 3a).

*Helminthope* cf. *psammobionta* Salvini-Plawen, 1991 (Smithsonian Institution No. SI-CBC2010KJ01_B05, a DNA aliquot): Collected 2010 at Carrie Bow Cay, Belize during meiofauna workshop of Smithsonian Institution (Station 4, ridge of outer reef slope, 15 m), from bulk sample of coarse subtidal sand, by Katharina M. Jörger, Jon L. Norenburg, Katrine M. Worsaae. Voucher preserved in 96% ethanol, tissue clipping sequenced (Figs. 2, 4).

*Ebala* sp. [ZSM Mol-20200025 (block 2b*7)]: Collected in 2017 at Yura, Sumoto, Awaji Island, Hyogo Prefecture, Japan (34°16’N, 134°57’E), from intertidal seagrass bed, by Sho Kashio. Voucher preserved in 80% ethanol after Formalin fixation, serially sectioned and used for 3D reconstruction of CNS (Fig. S3).

*Rissoella* sp. [ZSM Mol-20200026 (block 4b*7)]: Collected in 2017 at Kinchaku-jima Island, Uchiumi, Miyazaki Prefecture, Kyushu, Japan (31°44’N, 131°29’E), from upper subtidal coralline algae, by Yasunori Kano and Bastian Brenzinger. Voucher preserved in 80% ethanol after Formalin fixation, serially sectioned and used for 3D reconstruction of CNS (Fig. S3).

### DNA extraction, PCR amplification and sequencing

Full genomic DNA was extracted from clipped foot or mantle tissue using DNeasy Blood and Tissue Kit (Qiagen) or Macherey–Nagel Blood and Tissue Set and following the manufacturers’ instructions. Partial sequences of nuclear (18S and 28S rRNA) and mitochondrial (16 s rRNA and COI) markers were amplified using primers shown in Supplementary Table S1; see^69^ and^35^ for amplification conditions and other details. Amplicons were purified with ExoSAP-IT (Affymetrix) and then sequenced with Big Dye Terminator Cycle Sequence Kit 3.1 (Applied Biosystems) and amplification and sequencing primers (Supplementary Table S1). The reaction mixtures were analyzed on an ABI PRISM 3130xl sequencer (at AORI) or an ABI 3730 sequencer (at the Department of Biology Genomic Service Unit of the Ludwig-Maximilians-University Munich) after purification with Big Dye XTerminator Purification Kit (ABI). New DNA sequences have been deposited in the DDBJ⁄EMBL⁄GenBank with accession numbers LC631476–LC631487 (Supplementary Table S2).

### Phylogenetic reconstruction

For molecular phylogenetic analyses we selected 49 heterobranch species including one *Tjaernoeia* and two *Parvaplustrum* (Supplementary Table S2). The selection was made on the basis of covering the phylogenetic diversity of Heterobranchia and consistent evolutionary rates of all four targeted genes. The lower heterobranch genera *Architectonica* and *Ammonicera* were excluded from the main analyses due to their extremely long branches in a preliminarily tree. Dubious sequences, identified by BLAST searches and by careful comparison in the context of larger alignments, were excluded from succeeding analyses (see Supplementary Table S2 for notes on excluded sequences). Three species of Caenogastropoda were included in the dataset for outgroup comparison, resulting in a total of 52 taxa. The sequences of the four genes were aligned individually with MAFFT 7.182 using the L-INS-i strategy^70^; COI sequences were aligned as amino acids. Each aligned dataset was masked to remove alignment ambiguous sites on Gblocks Server 0.91b with all three options for a less stringent selection^71^.

Phylogenetic trees were reconstructed from single-gene and concatenated multi-gene datasets using the Maximum-Likelihood (ML) method implemented in raxmlGUI 2.0 (RAxML-HPC and RAxML-NG;^72–74^). Each gene and codon position was allowed to have different parameters, resulting in six partitions for the four-gene dataset. The RAxML-HPC analyses were performed using following commands: a rapid bootstrap analysis with 1000 replicates and search for the best-scoring ML tree in a single program run under the default GTR + G model, following the software manual. The RAxML-NG runs were carried out with the same setting to calculate transfer bootstrap expectation (TBE)^75^ values with 1000 replicates. The concatenated four-gene dataset was also analysed under Bayesian inference using MrBayes 3.1.2^76^. Substitution models used (estimated with jModeltest 2.1.10;^77^) were GTR + G for the 3rd codon of COI and GTR + I + G for all other partitions. Two parallel runs were made for 10 M generations with a sample frequency of 1000, using the default value of four Markov chains. The first 5000 trees for each run were discarded to make sure the four chains reached stationarity by referring to the average standard deviation of split frequencies^76^. The consensus tree and posterior probabilities (PP) were computed from the remaining 10,000 trees (5000 trees, two runs). Bootstrap proportion (BP) and TBE of ≥ 80% and PP of ≥ 0.99 were considered significant support.

The stability of clades was further tested in sensitivity analyses where taxa were selectively added or removed. The sequences of individual genes were aligned and masked by adding the long-branched clade of *Architectonica* and *Ammonicera* (55 taxa), or excluding rhodopid slugs or murchisonellid snails (50 taxa each), to generate three additional sets of four-gene matrices. These datasets were analysed in raxmlGUI2 with 1000 replicates to obtain BP and TBE values.

Divergence times were calculated from the four-gene dataset used in the main analyses (52 taxa) with the relaxed molecular clock model implemented in BEAST 1.5.4^78^. The tree was calibrated by setting ages for three nodes with reliable fossil records: (1) the split of Heterobranchia and Caenogastropoda by Early Devonian time (Gamma distribution, Shape: 1, Offset: 400, Scale: 13.34), (2) the first split in Euopisthobranchia by Early Jurassic (Offset: 190, Scale: 6.33), and (3) first split in Ellobioidea by Late Jurassic (Offset: 152, Scale: 5.07). See^35^ for the details of the calibration points and other settings for BEAST analysis.

### Microanatomical sampling and reconstruction

Ethanol-preserved specimens of *Tjaernoeia exquisita, Parvaplustrum tenerum* (Figs. 3, 4, Supplementary Fig. S3), and for comparison of the nervous systems, *Ebala* sp. and *Rissoella* sp. (Supplementary Fig. S3) were washed in 0.1 M phosphate buffer, decalcified using 3.5% ascorbic acid, stained in a solution of 3.5% Safranin in ethanol, then dehydrated in an ascending acetone series (70–100%), and finally embedded in Epon epoxy resin (except *T. exquisita* in Araldite resin). From the resin blocks, ribbons of serial semithin sections with a thickness of 1.0–1.5 µm were cut using a Diatome HistoJumbo diamond knife (Biel, Switzerland) with a Zeiss Microm rotation microtome (Jena, Germany)^79^. Sections were stained using Richardson’s stain^80^, sealed with Araldite resin and coverslips, and individually photographed using dotSlide software in combination with a semi-automated Olympus BX16VS microscope (both Olympus Soft Imaging Solutions, Tokyo). Photographs were resized, adjusted and changed to greyscale using scripts in Adobe Photoshop and imported into AMIRA 5.3 software (Visage Imaging, Berlin) for both manual and semi-automated realignment, segmentation, 3D reconstruction, and rendering of models. Presented images are surface renderings of histologically distinct structures or schematic drawings derived thereof.

## Supplementary Information

Supplementary Information.
