# Supplementary material for: Origin and significance of two pairs of head tentacles in the radiation of euthyneuran sea slugs and land snails
Source: Sci Rep. 2021 Oct 25;11:21016. doi: 10.1038/s41598-021-99172-5 (PMC8545979; doi:10.1038/s41598-021-99172-5)
Supplement: Supplementary file 1 — Supplementary Information. [file 41598_2021_99172_MOESM1_ESM.docx]

**Origin and significance of two pairs of head tentacles in the radiation of euthyneuran sea slugs and land snails**

Bastian Brenzinger*^1,2^, Michael Schrödl^1,3,4^ & Yasunori Kano^2^

*= corresponding authors: brenzinger@snsb.de, kano@aori.u-tokyo.ac.jp

Affiliations:

1SNSB-Bavarian State Collection of Zoology, Münchhausenstr.21, 81247 München, Germany. 2Department of Marine Ecosystems Dynamics, Atmosphere and Ocean Research Institute, The University of Tokyo, 5-1-5 Kashiwanoha, Kashiwa, Chiba 277-8564, Japan. 3Department Biology II, BioZentrum, Ludwig-Maximilians-Universität, Großhadernerstr. 2, 82152 Planegg-Martinsried, Germany. 4SNSB-Bavarian State Collection of Paleontology and Geology, GeoBioCenter LMU, Richard-Wagner-Str. 10, 80333 München, Germany. Correspondence and requests for materials should be addressed to B.B. (email: brenzinger@snsb.de) or Y.K. (email: kano@aori.u-tokyo.ac.jp)

**Supplementary information**

**Supplementary Table S1. Nucleotide sequences of primers used for amplification and sequencing.**

Locus Primer Sequence Direction Position^*1^ Reference

18S 18A1 CCTACCTGGTTGATCCTGCCAG Forward –21 to 0 Steiner and Dreyer (2003)

188f^*2^ GGATCTATTGGAGGGCAAGT Forward 535–554 Nakamura et al. (2007)

NS2^*2^ GGCTGCTGGCACCAGACTTGC Reverse 549–569 White et al. (1990)

NS5^*2^ AACTTAAAGGAATTGACGGAAG Forward 1143–1164 White et al. (1990)

189r^*2^ TCGGAATTAACCAGACAAATC Reverse 1310–1330 Nakamura et al. (2007)

1800r ATGATCCTTCCGCAGGTTCACC Reverse 1794–1815 Steiner and Dreyer (2003)

28S LSU5 TAGGTCGACCCGCTGAAYTTAAGCA Forward –24 to 0 Littlewood et al. (2000)

het3 CCCCAGTAACGGCGAGTGAAGC Forward 38–59 This study

400F^*2^ ACTCCATCTAAGGCTAAATA Forward 271–290 This study

ECD2S^*2^ CTTGGTCCGTGTTTCAAGACGG Reverse 790–811 Williams and Ozawa (2006)

D3m GACGATCGATTTGCACGTCAGAAT Reverse 1058–1081 Takano & Kano (2014)

16S 16Sar-veti GCCTGTTTAGCAAAAACA Forward –17 to 0 Kano et al. (2009)

16Sf50 GCCGCAGTACCTTGACYGTGC Forward 52 to 72 Kano et al. (2015)

16Sbr-H CCGGTCTGAACTCAGATCAYGT Reverse 409–430 Modified from Palumbi et al. (1991)

COI LCO1490 GGTCAACAAATCATAAAGATATTGG Forward –24 to 0 Folmer et al. (1994)

HCO2198 TAAACTTCAGGGTGACCAAAAAATCA Reverse 659–684 Folmer et al. (1994)

^*1^Position of primers on amplified sequences of *Parvaplustrum cadieni* (AORI YK#2783)

^*2^Sequencing primers

**References for Table S1**

Folmer, O., Black, M., Hoeh, W., Luts, R., & Vrijenhoek, R. DNA primers for amplification of mitochondrial cytochrome c oxidase subunit I from diverse metazoan invertebrates. *Mol. Mar. Biol. Biotechnol.* **3**, 294–299 (1994).

Kano, Y., Chikyu, E., & Warén, A. Morphological, ecological and molecular characterization of the enigmatic planispiral snail genus *Adeuomphalus* (Vetigastropoda: Seguenzioidea). *J Mollusc. Stud.* **75**, 397–418 (2009).

Kano, Y., Neusser, T.P., Fukumori, H., Jörger, K.M., & Schrödl, M. Sea-slug invasion of the land. *Biol. J. Linn. Soc.* **116**, 253–259 (2015).

Littlewood, D.T.J., Curini-Galletti, M., & Herniou, A. The interrelationships of Proseriata (Platyhelminthes: Seriata) tested with molecules and morphology. *Mol. Phylogenet. Evol.* **16**, 449–466 (2000).

Nakamura, K., Kano, Y., Suzuki, N., Namatame, T., & Kosaku, A. 18S rRNA phylogeny of sea spiders with emphasis on the position of Rhynchothoracidae. *Mar. Biol.* **153**, 213–223 (2007).

Palumbi, S., Martin, A., Romano, S., McMillan, W.O., Stice, L., & Grabowski, G. *The Simple Fool’s Guide to PCR, Version 2.0.* (Department of Zoology and Kewalo Marine Laboratory, University of Hawaii, Honolulu, 1991).

Steiner, G., & Dreyer, H. Molecular phylogeny of Scaphopoda (Mollusca) inferred from 18S rRNA sequences—support for a Scaphopoda–Cephalopoda clade. *Zool. Scr.* **32**, 343–356 (2003).

Takano, T., & Kano, Y. Molecular phylogenetic investigations of the relationships of the echinoderm-parasite family Eulimidae within Hypsogastropoda (Mollusca). *Mol. Phylogenet. Evol.* **79**, 258–269 (2014).

White, T.J., Bruns, T., Lee, S., & Taylor, J.W. Amplification and direct sequencing of fungal ribosomal RNA genes for phylogenetics. In *PCR Protocols: a Guide to Methods and Applications*. [Innis, M.A., Gelfand, D.H., Sninsky, J.J., White, T.J. (Eds.)] (pp. 315–322) (Academic Press, New York, 1990).

Williams, S.T., & Ozawa, T. Molecular phylogeny suggests polyphyly of both the turban shells (family Turbinidae) and the superfamily Trochoidea (Mollusca: Vetigastropoda). *Mol. Phylogenet. Evol.* **39**, 33–51 (2006).

**Supplementary Table S2. Gastropod species and sequences used in the phylogenetic reconstruction of heterobranch clades.** DDBJ/EMBL/GenBank accession number and length of sequences are shown along with the voucher of sequenced specimens. Bold letters indicate new taxa or sequences generated for this study. Accession numbers with an underline and an exclamation mark denote erroneous DDBJ/EMBL/GenBank sequences that were excluded from our phylogenetic reconstruction; those numbers with an asterisk indicate only one or two sequences were obtained from shown voucher specimen.

Clade Family Species 18S Length 28S Length 16S Length COI Length Voucher

Caenogastropoda Cyclophoridae *Aperostoma palmeri* DQ093435 1791 DQ279983 1951 DQ093479 519 DQ093523 657

Abyssochrysidae *Abyssochrysos melanioides* AB930376 1765 AB930325 2127 AB930403 475 AB930459 630 AORI YK#1482

Littorinidae *Littorina littorea* X91970 1831 AJ488672 1443 DQ093481 519 AJ622946 1199

Architectonicoidea Architectonicidae *Architectonica perspectiva* FJ917220 640 FJ917231 1536 FJ917251 429 FJ917282 577 EED-Phy-885

FJ917221 608 EED-Phy-885

‘Omalogyroidea’ ‘Omalogyridae’ *Ammonicera* sp.1^*1^ FJ917217 1990 FJ917233 1605 FJ917253 444 FJ917272 580 EED-Phy-568

*Ammonicera* sp.2^*2^ FJ917204 2013 FJ917234 1446 FJ917254 437 FJ917273 577 EED-Phy-567

Valvatoidea Cornirostridae *Cornirostra pellucida* FJ917215 634 FJ917225 3573 FJ917249 446 FJ917282 580 EED-Phy-884

Valvatidae *Valvata piscinalis* FJ917222 657 FJ917224 3627 FJ917248 472 FJ917267 580 EED-Phy-K388

FJ917223 826 EED-Phy-K388

Orbitestelloidea Orbitestellidae *Orbitestella vera* FJ917207 1793 FJ917239 1032 FJ917250 479 FJ917268 580 EED-Phy-518

Cimoidea Cimidae *Cima* sp. FJ917206 1862 FJ917228 3417 FJ917260 442 !FJ917279 (368) EED-Phy-904

*Larochella* *alta* !FJ917208 (1995) FJ917242 1139 FJ917261 449 FJ917280 580 EED-Phy-552

*Larochella* sp.^*3^ FJ917209 2273 FJ917230 3684 FJ917262 461 FJ917281 580 EED-Phy-553

**Tjaernoeioidea** Tjaernoeiidae *Tjaernoeia* sp. **LC631476 1750 LC631479 1027 LC631483 409 LC631486 658** AORI YK#2755

**Parvaplustridae** *Parvaplustrum* *cadieni* **LC631477 1793 LC631480 1061 LC631484 408 LC631487 656** SMNH-111919; AORI YK#2783

*Parvaplustrum* *tenerum* – – **LC631481 1057** – – – – ZSM Mol20021303/2 (B103)

Allomorpha Murchisonellidae *Murchisonella* cf. *anabathron^*^*^4^ !FJ917205 (1983) FJ917238 1108 FJ917259 439 FJ917278 580 EED-Phy-900

Murchisonellidae *Koloonella* cf. *nitidissima*^*5^ !FJ917218 (327) FJ917237 1098 FJ917258 422 FJ917277 580 EED-Phy-899

!FJ917219 (702) EED-Phy-899

Rhodopidae *Rhodope veranii* KY806807 1787 KY806816 1008 KY806798 455 KY806825 658 ZSM Mol20100300

*Helminthope* cf. *psammobionta* **LC631478** **537** **LC631482** **981** **LC631485** **408** – – SI-CBC2010KJ01_B05

Rissoelloidea Rissoellidae *Rissoella rissoaformis* FJ917214 1418 FJ917226 3681 FJ917252 403 FJ917271 577 EED-Phy-502

*Rissoella elongatospira* FJ917203 2175 FJ917232 1106 – FJ917270 577 EED-Phy-501

Acteonoidea Acteonidae *Pupa solidula* AY427516 1842 AY427481 1065 EF489319* 429 DQ238006 593 *EED-Phy-35

*Rictaxis punctocaelatus* EF489346 1387 FJ917243 2536 EF489318 365 EF489393 624 EED-Phy-454

EF489370 1071

Hydatinidae *Hydatina physis* AY427515 1804 AY427480 1087 EF489320* 420 DQ991932 14153 *EED-Phy-37

Ringiculoidea Ringiculidae *Ringicula doliaris* LC150577 1304 LC150580 1014 LC150582 2457 LC150582 2457 AORI YK#901

*Ringicula okadai* LC150579 1956 – LC150585 2470 LC150585 2470 AORI YK#1460

*Ringiculopsis foveolata* LC150578 1315 LC150581 929 LC150584 2520 LC150584 2520 AORI YK#1461

*Ringiculoides kurilensis* – – LC150591 438 LC150590 1273 AORI YK#2531

Nudipleura Bathydorididae *Bathydoris clavigera* AY165754 2064 AY427444 1383 AF249222 445 AF249808 599

Chromodorididae *Felimida krohni*  AJ224774 1887 AY427445 1064 AY345036 2647 AY345036 2647

Pleurobranchidae *Pleurobranchus peroni* AY427494 2022 AY427455 1120 EF489331* 443 DQ237993 593 *EED-Phy-436

*Tomthompsonia antarctica* AY427492 2105 AY427452 1156 EF489330* 440 DQ237992 593 *EED-Phy-435

Umbraculoidea Umbraculidae *Umbraculum umbraculum* AY165753 1843 AY427457 1069 EF489322* 439 AY345023 1360 *EED-Phy-51

Anaspidea Akeridae *Akera bullata* AY427502 1789 AY427466 1047 AF156127 418 AF156143 658

Aplysiidae *Aplysia californica* AY039804 1802 AY026366 3951 AF192295 422 AF077759 658

Cephalaspidea Philinidae *Philine exigua* HQ168425 1798 HQ168438 1036 HQ168412 420 HQ168450 624 ZSM Mol 20080752

Scaphandridae *Scaphander lignarius* EF489348* 1788 KC351544 1488 EF489324* 441 DQ974663 698 *EED-Phy-442

Haminoeidae *Haminoea hydatis* AY427504 1847 AY427468 1037 EF489323* 425 DQ238004 593 *EED-Phy-421

Sacoglossa Oxynoidae *Oxynoe antillarum* FJ917441 1998 FJ917247* 2411 FJ917425 487 FJ917483 593 *EED-Phy-723

FJ917466 1085

Volvatellidae *Volvatella viridis* HQ168426 1930 HQ168439 1074 HQ168413 435 HQ168451 657 AORI YK#890

Caliphyllidae *Cyerce nigricans* AY427500 1847 AY427463 1063 EU140843 441 DQ237995 593

Plakobranchidae *Thuridilla bayeri* AF249220 1847 AY427461 1058 DQ480206 434 DQ471271 618

Siphonarioidea Siphonariidae *Siphonaria pectinata* HQ659934 1785 DQ256744 2931 AY377627 438 AF120638 669

Glacidorboidea Glacidorbidae *Glacidorbis rusticus* FJ917211 1879 FJ917227 3493 FJ917264 454 FJ917284 577 EED-Phy-881

Pyramidelloidea Pyramidellidae *Turbonilla* sp. EF489351 1838 EF489376 1004 EF489332 473 EF489396 621 EED-Phy-526

Amphiboloidea Phallomedusidae *Phallomedusa solida* DQ093440 1816 DQ279991 2081 DQ093484 442 DQ093528 654

Hygrophila Physidae *Physella acuta* AY282600 1708 EF489368* 1011 AY651241 494 JQ390525 14490 *SMF 325459

Planorbidae *Ancylus fluviatilis* AY282593 1707 EF489365* 1042 EF489312* 427 AY282582 959 *SMF 325462

Acochlidia Parhedylidae *Microhedyle glandulifera* HQ168437 1796 HQ168449 1046 HQ168424 441 HQ168461 655 ZSM Mol 20081019

Pseudunelidae *Pseudunela marteli* HQ168431 1795 HQ168444 1032 HQ168418 438 HQ168456 655 ZSM Mol 20080393

Eupulmonata Discidae *Discus rotundatus* FJ917212 1854 FJ917240 1052 FJ917265 425 FJ917285 577 EED-Phy-607

Onchidiidae *Onchidium verruculatum* Y427522 1791 AY427487 1050 EF489316* 486 EF489391* 626 *EED-Phy-38

*Onchidella floridana* AY427521 1790 AY427486 1049 EF489317* 492 EF489392* 619 *EED-Phy-462

Ellobiidae *Smeagol phillipensis* FJ917210 1840 FJ917229 3399 FJ917263 425 FJ917283 577 EED-Phy-878

*Carychium minimum* EF489341 1739 EF489361 1061 EF489308 465 EF489386 663

Five specimens originally sequenced and identified by Dinapoli & Klussmann-Kolb (2010) as *Omalogyra fusca^*^*^1^, *Omalogyra* sp.^*2^, *Graphis* sp.^*3^, *Murchisonella* sp.*^*^*^4^ and *Ebala* sp.*^*^*^5^ have been re-identified by Warén (2013)^*3–5^ and the present authors^*1,2^.

**(a)**

**(b)**

**Supplementary Figure S1. Molecular phylogeny of Heterobranchia inferred from mitochondrial or nuclear gene sequences. (a)** Nuclear-gene tree based on combined nucleotide sequences of 18S and 28S rRNA genes (2636 characters). **(b)** Mitochondrial-gene tree based on 16S rRNA and COI sequences (993 characters). Tree reconstruction was performed in raxmlGUI 2.0 (RAxML-HPC); numerals on branches denote bootstrap proportions (in %). Note that all Parvaplustridae nov., Tjaernoeioidea nov., Mesoneura nov. and new clade Tetratentaculata were recovered in both nuclear and mitochondrial trees.

**(a)**

**(b)**

**(c)**

**Supplementary Figure S2. Sensitivity analyses for reconstructed phylogenetic relationships among Heterobranchia.** **(a)** Long-branched *Architectonica* and *Ammonicera* were added to original dataset (a total of 55 taxa), or either **(b)** rhodopid slugs or **(c)** murchisonellid snails were excluded from dataset (50 taxa each). Tree reconstruction was performed in raxmlGUI 2.0 (RAxML-HPC); numerals on branches denote bootstrap proportions (in %). Note that all Parvaplustridae nov., Tjaernoeioidea nov., Mesoneura nov. and new clade Tetratentaculata were recovered in every sensitivity analysis.

**
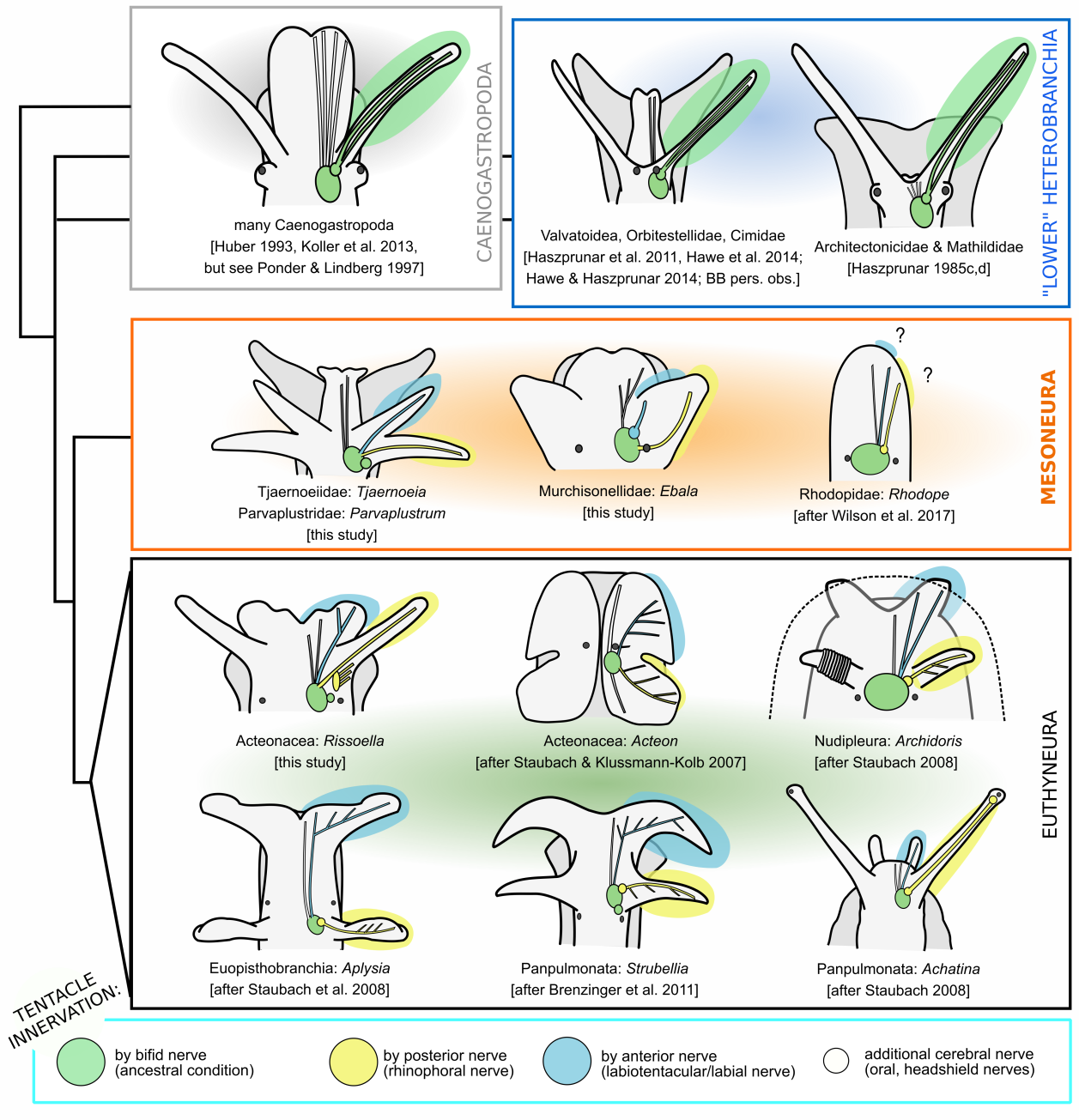
**

**Supplementary Figure S3. Expanded set of head morphologies for representative members of heterobranch subclades.** Our new findings lead to an evolutionary scenario of (1) splitting of one ancestral tentacle (as seen in top row) into two, each with one of the two nerve cords (Tjaernoeioidea, left in second row), and then (2) migration and specialization into the anterior and posterior tentacles of Euthyneura (third and bottom rows).

**References for Figure S3**

Brenzinger, B., Neusser, T. P., Jörger, K. M., & Schrödl, M. Integrating 3D microanatomy and molecules: natural history of the Pacific freshwater slug *Strubellia* Odhner, 1937 (Heterobranchia: Acochlidia), with description of a new species. *J. Mollusc. Stud.* **77**(4), 351-374 (2011).

Haszprunar, G. Zur Anatomie und systematischen Stellung der Architectonicidae (Mollusca, Allogastropoda). *Zool. Scr*. **14**(1), 25-43 (1985c).

Haszprunar, G. On the anatomy and systematic position of the Mathildidae (Mollusca, Allogastropoda). *Zool. Scr.* **14**(3), 201-213 (1985d).

Hawe, A., & Haszprunar, G. 3D-microanatomy and histology of the hydrothermal vent gastropod *Lurifax vitreus* Warén & Bouchet, 2001 (Heterobranchia: Orbitestellidae) and comparisons with Ectobranchia. *Org. Div. Evol.* **14**(1), 43-55 (2014).

Hawe, A., Parroll, C. & Haszprunar, G. Interactive 3D-anatomical reconstruction and affinities of the hot-vent gastropod *Xylodiscula analoga* Waren & Bouchet, 2001 (Ectobranchia). *J. Mollusc. Stud.* **80**(3), 315-325 (2014).
